# Dissertation on Psoas or Lumbar Abscess

**Published:** 1849-10

**Authors:** Frederick Hyde

**Affiliations:** Cortlandville, Cortland Co., N. Y.


### BUFFALO MEDICAL JOURNAL

AND

### MONTHLY REVIEW.

**VOL. 5.** OCTOBER, 1849. **NO. 5.**

### ORIGINAL COMMUNICATIONS.

ART. I.—*Dissertation on Psoas or Lumbar Abscess.* By FREDERICK HYDE, M. D., of Cortlandville, Cortland Co., N. Y. Read before the Medical Association of Southern Central New York.

[We have in a former No. of this Journal referred to this dissertation, and adduced some of the conclusions therein contained. We are happy to have it in our power to present our readers with the paper entire.—EDITOR.]

The importance of a medical or surgical subject is to be measured rather by the greatness of its character, than by frequency of occurrence. That a too exclusive devotion of the Physician and Surgeon's time may be given to the diseases of most common occurrence, is natural, as well as true. Such diseases as prevail most, will, of course, receive a corresponding share of his time, necessarily affording him more ample scope for observation, rendering his investigations more thorough, and his conclusions more philosophical.

It is perceivable, therefore, that a disease of rare occurrence may receive so sparingly of our time and study, as to lead to errors for which we may be justly open to censure. The disease suggesting the considerations presented in this paper has not been chosen with the expectation, or even hope, of making clearer its peculiar characteristics, or of adding any thing new to its system of treatment, but solely for the purpose of nducing some one or more to give to it such observations as its importance demands. With acute abscesses occurring in the exterior structures of the body, almost every practitioner is rich in observations; but can we say the same of chronic, and deep seated, or such as occur in visceral structures, and other hidden localities within the cavities of the body? If we can affirm this of some, are there not others where we are compelled to admit the uncertainty of our knowledge, and suffer the embarrassment incident to the employment of curative means, with the consciousness of an inadequate knowledge of the case under treatment. Those who have never been placed under circumstances of this character, are more fortunate than the writer.

A form of the disease, which seems peculiarly to have eluded the obser vations of the profession, at all events, one upon which an unaccountable silence has prevailed, is that to which particular allusion is now to be made, viz. *lumbar* or *psoas abscess.* This form of disease is noticed, and a brief space is occupied by its description, in all our elementary and standard works on surgery. But is it not true, that they have generally given to it neither time nor space, more than they have bestowed upon other diseases, far less intractable in their nature, and infinitely less hazardous to the well being of the human system?

Authorities all concur in the opinion of the serious character of the disease, and some of them, at least, in the general failure of remedial measures for its arrest and cure; and it is the more surprising that so little attention should have been bestowed upon a disease, however rare in its occurrence, confessedly so fraught with evil consequences to life as the one under consideration. During the period of our membership of the profession, our recollection has been unable to refer to a single article touching directly this subject, which has appeared in the medical periodicals of this country. Can it be said that its investigations have been carried to their ultimatum? Will it be claimed that it occurs so seldom as to make it a subject of small moment? Or will it be urged that in its very nature it is incurable, and, therefore, it will not recompense for its further study? We think the first inquiry is negatively answered in the meagre bibliography to be found upon this subject. And our opinion is, that whoever may urge the second objection, by a slight reference to the statistics upon this topic, furnished by practical members of the profession, will be compelled to witness the annihilation of his objection. To admit the third objection, viz. the unsuccessful practice in the disease hitherto, as a reason for limiting its further research, would be offering an insult to the spirit pervading the American medical profession.

Our observations have been confined to a territory of about thirty miles in extent, embracing Cortland County and its immediate vicinity, containing the average population of the farming regions of this State.

The period is thirteen years to which the writer is limited, and the number of cases coming under his cognizance during this period, is thirty, a portion of them, of course, accruing in the practice of professional neighbors.

This is a section of country that has rarely been visited by epidemics, and is remarkably free, too, from all causes that generate malarious diseases. The leading characteristic of its diseases, is phlogosis, especially of the thoracic viscera. It embraces no large manufacturing establishments, the principal avocation of its inhabitants being agriculture. The subjects of this disease have not been confined to any particular period of life; it occurred, however, in no one under 18 nor over 70 years, while the majority of the patients were from 40 to 50 years old.

It is undoubtedly true of this disease, as of some others, that it does not prevail to the same extent in all parts of the United States. From all the statistics we have been able to collect, it is clearly shown that in this State, as well as in some portions of New England, the disease is more common than in some of the more southern portions of our country. The experience of our northern practitioners, arrayed with that of the more southern brethren, furnishes abundant proof upon this point. When such statements as the following are taken into the account, made by Prof. Gibson, of the University of Pennsylvania, in which he declares he has seen only twelve cases of lumbar abscess, in a period of twenty-three years, our conclusions must inevitably be in favor of the infrequency of this disease in the south, compared with its prevalence in the more northern States. If this opinion be not correct of Philadelphia, and its vicinity, how are we to reconcile the statement of the eminent professional teacher whose name we have here introduced, whose field of experience has been so extensive, with the reports of private practice in this section of our country, which show a larger number of cases, occurring, too, in a shorter period, and upon a far more limited field for observations in practical surgery.

Of the whole number of cases to which reference is made in this paper, five only occurred in females, one of which showed the disease as early as the eighteenth year; three between the thirtieth and fortieth, the fifth dying in her fiftieth year. Of this class of patients but one exhibited the physical conformation recognised as the scrofulous diathesis. Of the twenty-five males who had the disease, two only gave evidence of possessing the strumous habit; while the remaining number were of vigorous constitution, affording no suspicions either of the scrofulous or tubercular predisposition. The period of the disease varied from two months to seven years. But one case only was so brief in its career as to be limited to the first named period, while three only were extended through the latter. The majority of the cases were of about three years' duration. It should be understood, that the period here assigned to the disease, includes only its progress from the earliest date of its specific manifestations presented to our notice, to its termination. The earliest symptoms that mark an attack of psoas abscess so very rarely come under our cognizance, as to leave much obscurity upon its early diagnosis. The ordinary period at which professional advice is first sought in this disease, is one so uniformly exhibiting the stage of structural change, as to make the diagnosis an insecure basis for any other than an unsuccessful treatment. Hence, the propriety of the concurrent opinions of some experienced surgeons in the general failure of the treatment given to lumbar abscess. That a great want of identity in the signs of this disease does exist in different cases, even at the stage when our opinions are demanded, is a conclusion, we think, to which every practitioner must have arrived, whose observations have been directed to this subject. To-day a patient comes to us with a well developed physical organization, of good general health, for advice in reference to a lameness of the hip. He says it has troubled him for months, dating its commencement soon after some extreme exercise of body, or some palpable exposure to atmospheric changes. He can stand very nearly erect, walks with a slight aberration of gait, which he says is only owing to a little stiffness ; the parts are free from tenderness and swelling; no discernible disparity in the length of the extremities; no tenderness in the lumbar region; appetite tolerable ; pulse nearly natural; he calls it rheuma tism, and so do we, and let him pass. Two years later he returns to us, with haggard features, muscles atrophied, thigh permanently upon the pelvis, saying that he has come to see to us concerning a swelling upon his thigh. He exposes the limb, and it presents us with a pale femoral tumor about the size of an orange. We examine it; fluctuation is palpable, and, however unwilling, we are compelled, in obedience to our judgment, to admit the existence of psoas abscess, and that, too, in an advanced condition. Again, a young gentleman of healthy look comes to us, declaring that at a certain time, a few months previous, after performing an extraordinary feat at lifting, he was exposed to a cold and severe storm, since which he has had slight pain and soreness in the back, and on gluteal region, always increased after taking hard exercise. General health tolerable. We examine him, tell him his disease is lumbago, give him a temporary prescription, and he goes to his accustomed avocation. Four years afterwards we are called to the same patient, with particular reference to a tumor occupying the right iliac region. We find him emaciated; indeed, he exhibits most clearly all the hectic phenomena. Our diagnosis establishes, beyond a doubt, the purulent character of the contents of the tumor, and leaves but the slightest uncertainty that its origin was in the psoas region.

In the next instance, our advice is asked in the case of a young lady of eighteen years, in whom we recognise, at once, all the physical charactertics of the scrofulous diathesis. She says, a year ago she contracted a severe cold, which was followed by rheumatism of the extremities. It subsided so far in the course of a few weeks, that she recovered the use of all her limbs, except one of the lower extremities, which continued slightly lame, but exempt from pain for some months, when she began to have dull and constant pain in the back and in the region of one hip. Her menstruation was arrested at the time of her exposure to cold, and has not returned She now has poor appetite, impaired digestion, quick pulse, and much general nervous irritability. In our investigation we fail to find positive proof of structural disease, but from a slight tenderness evinced by pressure upon some portions of the vertebral column, we give to her case that encyclopedial name, *spinal irritation.* A few months thereafter we are invited to visit this lady again. To our great surprise we find her confined to the horizontal posture, and helpless, with one lower extremity drawn up; cannot endure the least effort to extend it, has a constant diischarge from the thigh, at or near the saphenous opening. The whole aspect of the case is now decidedly hectic. Should we ever recover from the confusion of mind incident to a consciousness of having wrongly diagnosed a case, already far advanced towards a fatal termination, we shall be willing to acknowledge, for once at least, that upon the subject of psoas abscess, we are not quite so wise as we should choose to be. A gentleman aet. 40 years, who has always had good health, sends for us to see him in a sudden attack of severe pain in the gluteal region. He says he has had very slight pain in the same place occasionally for a year, but never to amount to suffering, or inability to exercise moderately until now. There is now slight pain through the lumbar region; he has loss of appetite and moderate fever. No external swelling of parts. The pain soon subsides under the use of anodynes, and, with a little corrective treatment, he is soon able to resume his labor, but occasionally has similar attacks of pain in nearly the same location. In the course of a few months, his general health shows a decided deterioration; the pain, although confined to the same region, instead of remaining paroxysmal, is now continuous. He is now compelled to keep his room. We see him again, re-investigate his case, pronounce the hip-joint structures, and all others affording suspicion of morbid action, exempt from lesion, and treat the case as sciatica, or general neuralgia of the neighborhood. A few weeks more elapse, and the patient invites our attention to an unusual projection at quite the lower extremity of his body. We examine, and find, near the anus, a pale swelling, about double the size of an almond. It affords unequivocal fluctuation, and we have very little doubt that its contents are purulent. In a few days more, it ruptures spontaneously, and discharges profusely a whitish purulent matter, which after a few days becomes moderate in quantity, but still constant; irritative fever supervenes, he wastes rapidly, and in three months from the time the discharge first took place, he dies. Post mortem examination only confirms the revelation already made in the phenomena of the pointing, locality, character, and persistent discharge of the matter, that the patient died of *psoas abscess*. Our memory, refreshed by a review of the individual cases of this disease which have come under our notice, is unable to arraign even two that did not present a discrepancy of phenomena in their earlier stages, to such an extent as to embarrass very much their early and correct diagnosis. This want of sameness in the symptoms of the earlier periods of the disease, is frequently much in the way of correct conlusions as late as the external pointing of the abscess. Even with all the assistance afforded by the external development of a tumor containing fluid, (to say nothing of the uncertainties of its character,) the writer thinks he has experienced much perplexity in answering satisfactorily the question, what is the origin of the purulent accumulation? or where did the disease commence its original devastations? If it has never been the misfortune of others to have answered this question incorrectly, it is the duty of the writer to acknowledge his unfortunate dilemma, in this respect, once and again. Illustrative of our position, that the want of more accurate knowledge of the early symptoms, no less than the dissimilarity in the subsequent developments, and the great range of period observed by the disease, render an early diagnosis not sufficiently definite for the basis of a radical plan of treatment, is adduced the history of the following cases:—

G. B., aet. 60 years, of sound constitution, capable of, and had endured much muscular fatigue for years ; was residing in the midst of an endemic typhoid fever. He became a subject of the disease, (the prevalent fever,) in whom it passed as with the same degree of severity that marked its progress in other patients in near proximity to himself. He seemed to have reached and fairly entered upon the convalescent stage, when he was attacked with pain in the abdomen, at first rather moderate, but in a few days becoming severe, persisting obstinately in opposition to all the remedies used for its relief. Abdomen slightly distended, with extreme tenderness of the umbilical region, bowels readily moved by aperients, aversion to food, tongue slightly coated, with red surface, and pulse from 120 to 130. Pain in the abdomen continued; tenderness more diffuse; did not complain of pain in the back ; and now all the features of the case were assuming more and more the hectic character for about five weeks, when a tumor, about five inches at its base, its form nearly conical, was discovered to the left of the spine, in the lumbar region. No discoloration of integuments covering the tumor ; fluctuation distinct; which circumstances gave the strongest suspicions of the existence of advanced lumbar disease. A valvular opening was made near the base of the tumor, which was followed by the discharge of a pint of pus, with some floculi, when the opening was closed, and adhesion allowed to take place. Constitutional symptoms continued nearly the same for two weeks, when a re-accumulation showed itself at the same point as before, with so much pressure as to demand another evacuation, which was made in the same manner as in the first instance, but was not followed by adhesion of the opening ; a constant discharge kept up, general irritation increased, exhaustion and death occurring in a few days.

B. F., aet. 40, of good physical organization ; occupation carpenter. At the raising of a building seven years before, he made an extraordinary exertion at lifting, upon which he immediately after experienced some new and uncomfortable sensations in the back, and he afterward walked some distance in a storm of rain. For several days he was confined to the house on account of a lameness, rather than pain in his back, from which in a few weeks he so far recovered as to resume his labor. He found ever afterwards that certain kinds of exercise caused a return of the lameness, attended with more pain at each recurrence, and extending through the region of the right hip, but ordinarily he was not obliged to suspend his labor more than a week at a time, for about five years. He now began to have more pain and lameness in the right hip, said his back was well, still laboring at short intervals for six months more, but at no time, during this last period, was he known to stand erect. He was now compelled to suspend pretty much entirely his labor, complained still more of the hip, and occasionally of severe pain in the thigh of the affected side, insisting that his back was well, and it indeed evinced no tenderness, by considerable pressure applied at any point upon the lumbar, or any other portion of the vertebral column. His general health was now so much impaired that he was unable to leave his house, had more pain in the hip and the thigh, the latter more flexed upon the pelvis, and irritative fever constantly increasing. About this time a pale fluctuating tumor appeared near the middle and upon the anterior aspect of the thigh. It was opened, and a profuse purulent discharge followed, with considerable relief of pain for a few weeks. The discharge continued, appetite rather better than before the opening, and patient still urging that his back was sound, it exhibiting no external evidence to the contrary. Thigh still more drawn up, yet able to move himself daily from place to place in his room.

He went on with scarce a single day's remission of the discharge, gradually emaciating, suffering but little severe pain after the opening for about ten weeks, when he became unable to leave his bed. After several weeks' confinement to the horizontal posture, he referred most of his pain to the back, and, some portions of the time, it was extreme. The purulent discharge continued, emaciation became extreme, prostration of the powers of life more and more, until death occurred, about the ninth month from the time the abscess was opened, and a little more than seven years from the first attack of lameness in the back.

In neither of these two cases was a post mortem examination made. At a certain stage of each, no want of coincidence of opinion existed among all the different medical gentlemen who were conversant with them, as to the identity of disease and structure involved in the two cases. But on a review of all the marked characteristics developed in their progress, we find them to be equally as discrepant, as the period belonging to each. As corroborative evidence in favor of our position, we next cite two cases in which examinations were made after death:—

The first, a young lady of eighteen years, had her menstruation arrested by what seemed some accidental cause, which was followed by more severe and protracted pain throughout the abdominal region than ordinarily ensues upon the accidental obstruction of this discharge. She became a little fe[illegible]erish, bowels costive, the menstruation only returning at irregular perioas, [illegible]t each recurrence more imperfect in its effort. Under [illegible]tate of things [illegible] progressed about a year, never in the time enjoying entire exemption from the abdominal pain, always aggravated by exercise, especially walking, and a gradual and constant increase of emaciation. At the end of the year she was unable to keep the erect posture, from the severity of the pain. The abdomen was preternaturally full, the pain more intense ; and some of the distinctive indications of the hectic state were now clearly exhibited. She lingered, without any palpable external developments, about four months subsequent to her confinement to the bed; when, suddenly, the abdomen was much increased in size, delirium came on, and death occurred in a few hours.

Dissection, 20 hours after death, showed abscess upon either side of the lumbar spine, occupying the psoas region, the pyogenic membrane containing the purulent collection upon the right side having ruptured, its contents had escaped, and were diffused among the abdominal and pelvic viscera, while that upon the opposite side had preserved its integrity. About three pints of pus, containing some floculent shreds, were taken from the abdominal cavity, which had escaped from the abscess upon the right side, while the cyst upon the other side contained nearly a pint. The extent of the sac which had ruptured, was from the superior origin of the psoas magnus, down the course of the muscle, taking the track of the femoral vessels to the point of their emergence beneath Poupart's ligament. Not a vestige of the psoas muscle was to be found. The sac upon the left side was much less extensive, some portion of the muscle towards its inferior extremity was still traceable to its tendon of insertion. Vertebræ sound.

The next case occurred in a lady who, up to the end of the forty-first year of her life, had enjoyed good health, had been accustomed to the severest muscular efforts, and had borne several children. She was attacked with pain referred to a point indicated at the second and third lumbar vertebræ, which, by paroxysms, was severe, but never entirely abated. Under these circumstances she gave birth to her last child. The labor was no more than ordinary. On the fifth day subsequent to delivery she was seized with pain at the ala of the pelvis of the left side, which continued several weeks, and was followed by pain in the right ala, resulting, in a short time, in considerable pelvic distortion. She had constant but not severe pain in the back; could not assume the erect posture, but called her general health tolerable. About this time, being two years from the first attack, she was seized with violent paroxysmal pain in the abdomen, most severe in lower part, which continued about two weeks, when she began to refer its situation more to her back, and, at times, it was lancinating. She complained much of numbness upon each side of the lumbar vertebræ. Three months from this last attack of pain, anterior curvature of the spine was distinctly marked, the point of angle seeming to be at the junction of the first and second lumbar vertebræ. Her general health still allowed her to superintend the domestic affairs of her family, but she was subject to constant moderate pain in the lumbar region, but affording very little evidence of tenderness to almost any amount of pressure applied at any point upon the spine. Occasionally, after having more than her ordinary exercise of body, she would get a slight return of the spasms, which she would relieve by anodynes.

In this general condition she passed on about a year and a half, the only obvious change being the increase of the curvature, and its lateral inclination. She now called the writer's attention to a swelling, which, she said, appeared upon the upper part of the thigh several months since. Its commencement was attended with severe, and, as she called it, "bursting pain," which continued about four weeks, after which its severity was much less. She stated that since the swelling appeared, her back had been much better, and all she now desired was, that the tumor might be healed, the crook in her back she cared nothing about. The tumor had no discoloration, was smooth, about four inches in length, of oblong shape, projected two inches above the surface of the limb, occupied the crural region, and presented all the characters of an adipose tumor. During the first few weeks after she discovered it, it lessened in size, but never entirely disappeared, even in the lying posture. At this time, change of position made no difference in its size, yet it was a little softer in the horizontal, than in the erect posture. The thigh was still more flexed upon the pelvis. She went on another year, being able, the greater part of the time, to walk, though with much inconvenience and increase of suffering. Spinal distortion increased; tumor trebled in size during the last year; fluctuation not positive. At this period a general hydropic condition of the system appeared, particularly in the form of ascites. The dropsy subsided under the use of the ordinary remedies, and, for a few months subsequently, the tumor diminished considerably in bulk, with some improvement in the general health. The pain soon returned again, in the lumbar region with more severity, and extending more than ever down the limbs.

The swelling immediately began to increase, and continued to grow, until it extended itself from Poupart's ligament to within four inches of the bend of the knee, varying from four to six inches in breadth, and four in depth, producing much pain and uneasiness in the entire extremity. The coverings of the tumor were quite tense, fluctuation perceptible, and at two points towards its lower end the walls of the sac were quite thin; and if left to itself would open spontaneously soon. Under these circumstances a puncture was made in the tumor, and between five and six pounds of a greenish yellow, sero-purulent fluid, containing shreds of a curdy substance, discharged, with much relief to the patient. A compress and roller were applied to the limb, and adhesion took place throughout the greater portion of the sac occupying the thigh; and for a few weeks her health seemed a little improved. An opening continued in the thigh, from which a constant discharge took place. Emaciation now became extreme; lower extremities œdematous, and exasperation of all the hectic symptoms, followed by death, between the seventh and eighth month from the time the abscess was punctured. Dissection showed obliteration of the sac in the thigh, except in a small canal reaching from the external opening up to beneath Poupart's ligament; but from this point upwards the pyogenic membrane was continuous to the origin of the psoas magnus, except along the sides of all the lumbar and one dorsal vertebræ, where at various points it was entirely wanting. No traces of either the psoas or iliac muscles were to be found upon this side; not even a vestige of them, at the different points of their respective origins. The two superior lumbar vertebræ were carious for about one-half the surface of their bodies, upon the side of the abscess; the three inferior were considerably roughened at various points, upon the same side, as well as the last dorsal. Both anterior and right lateral curvature existed. Parietal and visceral structures normal.

In examining the history of these two cases, we are scarcely able to find a characteristic symptom of one, (except it be quite in the last stage,) belonging to the other, and yet, upon dissection, it is clearly demonstrated that they both labored under, and died of the same disease; the latter, it is true, exhibiting the additional complication of disease of the vertebral structure.

Do not these results afford quite positive support to the position assumed in this paper, that the contrariety of circumstances accompanying the disease in different cases, after they come under the notice of the surgeon, renders a diagnosis too precarious to admit the timely interposition of adequate means to prevent a fatal termination of the disease in a majority of cases? The first case was closely investigated, during its progress, by several physicians and surgeons of acknowledged professional skill and acumen, but no one of them ever declared the opinion, until after death, that the patient was laboring under *psoas abscess.*

The second was subjected to the investigation of a greater number of medical gentlemen than any other of the kind ever coming under the notice of the writer; many of whom, it is but justice to allow, were of the first eminence in this country; and even with all the aid afforded by the presenting tumor in the femoral region, it was quite as often diagnosed some other, as the disease revealed upon dissection.

Is the disease necessarily scrofulous? That in its progress, at some of its stages, it may be characterised by many of the circumstances peculiar to this affection, cannot be denied, any more than that the disease sometimes occurs in that condition of the system termed the strumous habit. It will no doubt be inferred that the writer's opinion upon this question, based as it is upon conclusions derived from data afforded by a given number of cases, would be in the negative. That it is so, he is frank to acknowledge, conscious, at the same time, that his views may be at variance with those entertained by some of his brethren. He only claims legitimacy of conclusion as derived from a specific group of cases, limited to a specified territory, carefully noted by his own observation. But it may be urged that he has admitted a certain proportion of the group to have exhibited the scrofulous habit. This he does not deny, and he here re-asserts that the balance of the number were remarkably free from tendency to constitutional disease. He believes, too, that the patients all had the same disease, an opinion which, so far as dissections were carried, was amply sustained, both in identity of original structures involved, and results of the diseased processes.

Our conclusion, then, is, that the disease, in the instances here cited, originated in a concurrence of accidental causes, rather than in any predisposition to it in the physical organization; at the same time, believing that a patient, having the scrofulous diathesis well stamped upon the organism, when exposed to the same amount of causes, would receive the disease with more readiness, having less resisting power than one of sound bodily habit. Again, we urge as an argument against the necessary dependence of this disease upon the scrofulous diathesis for its production, the age at which it ordinarily manifests itself. It will be recollected that the data upon which our conclusions rest, show that a decided majority of the cases occurred at too advanced periods of life to allow the belief that their development was traceable to an identity with the scrofulous predisposition. We have no doubt it will be conceded that scrofula is a physical condition peculiar to the adolescent periods of life, and if not radically changed previous to, or favorably modified, certainly as late as the period indicated by the age of the youngest patient of our catalogue, its character is positively evinced, either by the disastrous ravages already made, or in the death of its victim.

What are the structures originally involved in this disease? If we consult authorities upon this question, with all deference to their wisdom, we shall be compelled to satisfy ourselves with none other than vague conclusions as to the structure first subjected to the morbid action. While one declares that it commences in the sheath, another tells us that the substance of the psoa muscle itself is its original seat; a third says it always begins in the vertebræ; a fourth that the variety of the disease which is fatal, invariably has its origin in the lumbar vertebræ. Others, choosing much broader foundation for their opinions, say the disease of the vertebræ may be either a *cause* or an *effect* of *lumbar abscess.*

The illustrious Physic said he never met with a case of psoas abscess, in America, unconnected with disease of the spine. The results of dissection in nine cases of the disease, seven of which were made by the writer, with especial reference to the original seat of the disease, (the examinaion of the remaining two being made by a professional friend who kindly furnished us with notes of the same,) furnish conclusive evidence for the exemption of the vertebral tissues, not only from original, but even from consecutive implication in this disease.

But one only of the cases examined, exhibited the slightest evidence of lesion of the vertebræ; and that one most clearly connected with curvature of the spine, the details of which have already been given.

True, these results only furnish positive proof in the cases examined, but it cannot be denied that they afford the strongest presumptive evidence in favor of the primary seat of the disease being found in some other than the vertebral structure, in at least a majority of the cases. In support of our views upon this point, we think we may not only urge with much propriety the extension and peculiar origin and insertion of the psoas muscle, but its function. When we reflect that its origin is muscular, and from the bodies and transverse processes of all the lumbar and two inferior dorsal vertebræ, assuming the best possible form to give it strength, terminating in a powerful tendon, which takes hold of the most favorable point of the femur for producing and controlling its motions, while, from its origin, it is the controlling power of the position, and most important movements of the body as connected with the lower extremity—we connot fail to see its great liability to injury from the varied emergencies to which the body is exposed, which even though it be ever so slight, from the impossibility of affording the requisite quietude to the injured muscle, while the general motions of the body are indulged, may thus insidiously lay the foundations of disease, ultimately destroying the muscle. It seems to us that the fact of so large a proportion of our group exhibiting, upon dissection, entire exemption of the spine, (a single exception only existing,) must be explained upon some other ground than mere coincidence. The writer is far from questioning the correctness of the conclusions of others as to the original seat of this affection, only making his deductions, as already stated, from the limited range of his own experience. In obedience, therefore, to his present convictions, he cannot entertain any other opinion than such as assigns, as the original structure of the morbid process, either the psoas muscle, or its immediate envelopes, in the majority of instances, and that the cases which show an alteration of vertebral structure, are produced by an extension of the original disease of the muscular tissues.

*Prognosis.*—From the allusions already made to the imperfections of diagnosis in this malady, an unfavorable prognosis will doubtless be inferred. We regret that our experience will not allow us to indulge in other predictions of the termination of this disease, than such as afford so sad a commentary upon our curative efforts. However much it may conflict with the experience of others, or to whatever extent it may mortify our own professional pride, a scrupulous regard for truth demands of us the admission, that we never have witnessed a single example of complete recovery in a well marked case of this disease. That some cases have been reported as recovering entirely from the disease, we are well aware, and it illy becomes us, with our narrow means of observation, to question the truth of such statements. But, on the contrary, do we most cheerfully congratulate him whose efforts in the treatment of the disease in question will enable him to declare an experience which will afford him better reasons for self gratulations than the writer feels at liberty to indulge. He can say that he has seen several cases which, for a term of months, and one, even, that for a period of almost three years, gave much promise of permanent recovery; all of which had discharged purulent matter, but which in each case was suspended a sufficient length of time to allow strong hopes of a final cure; but, in every instance, the disease returned with exasperated character, ending fatally at last.

He feels confident. too, that he has witnessed cases presenting so many of the symptoms of this disease, as to allow them to be called such, when, in truth, they were some other malady. The following is a case in point: B. F. E., aet. nearly 70 years, had for several months the usual premonitions of psoas abscess; after which a tumor presented on one side of the spine in the lumbar region. His general health became bad, and in a few weeks the swelling had attained the size of a coffee-cup; coverings pale, fluctuated distinctly; and had all the ordinary characteristics of a cold or chronic abscess. It was pronounced a lumbar abscess, and was treated as such by valvular opening, etc. It discharged freely of pale pus, containing curdy lumps, for nearly two months, but, gradually lessening, it finally ceased, and the opening closed. His general health became tolerably good, and he died, two years subsequently, of organic disease of the heart, as demonstrated by dissection, neither the psoas muscles, or spine, giving the least evidence of ever having been diseased.

H. L., aet. 33, of good general habit; for two years had been subject to constant but not severe pain in the right hip region; the thigh upon the same side for the last six months had been slightly flexed upon the pelvis; he has now a tumor as large as a middle-sized orange, situated immediately below Poupart's ligament, which he first noticed six weeks since; is not discolored, fluctuation doubtful; general health moderately failing. In four weeks more, the tumor had trebled its size, and fluctuated distinctly. It was opened, and discharged two pounds of laudable pus; opening did not close; the discharge continued daily, but lessened gradually in quantity for a few weeks, and ceased. He had but slight pain, and was still able to take moderate exercise. He now began to have some pain in the other hip, of the same character which he had experienced in the first. This, too, was considered a case of psoas abscess. About this time he was attacked with severe pain in the upper part of the abdomen, which was soon followed by vomiting, and he died on the fourth day, having had well marked symptoms of total obstruction of the intestinal canal, which was explained, on dissection, by the discovery of a tumor within, and near to the lower end of the duodenum; which occupied the entire calibre of the intestine. The iliacus internus muscle, upon the side of the abscess, was totally obliterated; psoas muscle sound; no traces of the sac; but entire closure from the point at which the pus had been discharged, up to the pelvic cavity. Upon the opposite side a gill of pus was found, lying upon the belly portion of the iliac muscle. Spine sound.

The first case was denominated, treated, and cured as psoas abscess, all in good faith, and yet, upon dissection, it was clearly proved that no such disease had existed.

The second instance gave no less positive assurances of the existence of the disease in question, but when subjected to the same tests, although not developing the same pathological condition as the first, showed both psoas muscles ,and spine, equally exempt from all disease. While we feel warranted in uttering the belief that some of the reported recoveries from this disease were rather supposed than real, and that others, from the close semblance of external signs, have been falsely diagnosed; we are frank to admit that, here and there, a case has been cured; receiving the appropriate treatment of its incipient stages, while no suspicions were ever entertained of the real nature of the malady. Take, as an example, a case of what appears to be a severe lumbago, in its very earliest existence; where a decided anti-inflammatory treatment is promptly instituted, with an entire compliance, for the requisite period, with the injunction of perfect repose; the morbid process being an inflammation of the psoas muscles, or their coverings, or both, instead of their more exterior parts, and we shall see subdued a morbid action, which, if allowed to take its legitimate course, would result in lesion of the muscle.

As already intimated, our experience, hitherto, will not justify us in the recommendation of any plan of radical treatment for this affection, with that confidence which ought to inspire him who assumes the responsibilities incident to the treatment of so formidable a disease. True, we might urge, under this head of our subject, sufficient in the way of treatment to afford us the self-complacency of having the acknowledged standards to rest ourselves upon, were it not for the sad misfortune under which the writer labors of having pursued the same with so unfavorable practical results. We are told by one authority that the anti-phlogistic treatment is indispensable during the early stages of the disease; but he has failed to indicate to us the least evidence by which we may discover the condition of the case to which he would apply such treatment. Again, we are advised to resort immediately to the system of counter-irritation, whenever the spine is the seat of the disease, without specifying a single distinctive characteristic to prove the existence of morbid action in this structure, during any thing like an approach to its early stages. Next, comes a portion of treatment to which, with very few exceptions, general assent is given; a resort to which is called for, by the occurrence of more tangible proof, the external pointing of the abscess, provided the nature of the same can be determined, a point not always easily established, even though the surgeon possess the *tactus eruditus*—from the variety of localities or points at which it may show itself, as well as the uncertainty as to the precise character of the contents of the tumor. We refer to the Abernethean method of evacuating the contents of the abscess, having for its object the adhesion of the walls of the sac. The writer regrets that the results of his observations will not allow him to ascribe to this practice a single instance of permanent recovery in support of its claims as a curative measure. We do not hesitate to avow, frankly, that this plan of treatment was an achievement in practical surgery of the highest value, well worthy of its great author, but deriving its importance more from its competency to soothe the sufferings, lengthen the period of life, and make tolerable the condition of the patient, than from its potency to eradicate the disease. Unfortunately it has happened to the writer that in more than half the cases which he has treated himself, or seen treated by others, upon this plan, adhesion has never followed the first opening. This is a circumstance of necessary occurrence from two causes—the advanced stage of the disease at which external pointing occurs, and the length of time suffered to elapse after the appearance of the tumor before an opinion is demanded; and, perhaps, with equal propriety we may add a third cause, viz. the uncertainty of its character, even after the pointing has taken place.

Our opinion is, even on the ground of a palliative treatment, (and this is certainly the basis upon which we choose to place this, with all other methods of discharging the contents of genuine psoas abscess,) that the plan advised by Prof. Miller, of Edinburgh, is decidedly preferable to any other. See Miller's Principles of Surgery, page 152. It will readily be seen that an opening, after this manner, will be altogether more likely to allow adhesion to follow the evacuation, and incur far less liability to ingress of air; but more especially will its superiority be appreciated in those cases in which the parts are much attenuated previous to the opening being made. But to dwell upon the details of treatment only applicable to such stages of a disease as are marked by the extent of structural change which affords the first and only positive proof of the existence of the one we are discussing, seems to be an useless labor, beyond what we have assumed to be palliative in its nature. If the original seat of this disease be in the psoas muscle and its coverings, (and it will be recollected that, according to our data, we were compelled to adopt this opinion,) and suffered to progress until manifested by the presentation of purulent collections at various points exterior to the abdominal cavity, previous to the use of remedial measures, the great destruction of tissues which make up so important a part as this muscle, indicated in such pointing, most clearly puts at defiance all rational hope of success with any radical plan of treatment. Adopting this view of our subject, which we consider is abundantly humiliating, it becomes us to go back, and extend our researches, for the purpose of accumulating more available diagnostic evidence of the incipient morbid phenomena, if we would avail ourselves of a system of treatment that shall triumph over this disease.

We ask, what could afford higher gratification to the surgeon than to possess a knowledge which would enable him to detect the first morbid impression in the production of so serious results as follow this disease? Who does not feel fully the important demand of more positive pathognomonic proof of the very first onset of this disease; desiring to see his profession freed from the imputation, so legitimately charged upon it, of being fruitless in its curative resources, when applied to the ailment in question! Who comes to the treatment of psoas abscess after it has assumed those external palpable signs of great changes already wrought, without a faltering hand and discouraged heart, in view of the inevitable fatality which is to fix the seal upon the practical efforts of his skill! Have we, then, any alternative, but, by our more thorough investigations, to secure more reliable and positive information of the symptoms belonging to the first diseased action that exists in the case?

We therefore inquire if there may not be an earlier group of symptoms accompanying its stage of innovation that has hitherto eluded our notice? Is not phlogosis of the primary structures involved in the disease, marked by symptoms which would clearly distinguish it from lumbago, or any other morbid affection of exterior structures? Is not the non-fulfilment of the functions of this muscle, in reference both to the movements of body and extremity, peculiar to the disease? Shall we not find that, however slight the inflammation may be, it will be attended with a certain degree of fixture of the femur upon the the body, marked only by the slightest angle of flexure, and yet all attempts to resume the natural relative situation of extremity and body, by extension of the former, if successful, produce pain throughout the course of the muscle, by putting it, while in its excited and tender state, so much upon the stretch? May we not discover, upon carefully watching the locomotive efforts of the patient, long before he suspects himself disabled, beyond what he, as well as ourselves, have called rheumatism, certain reliable signs of impaired function of the muscle which we have chosen to consider the foundation structure of *psoas abscess*? Will not our suspicions be equally strong by close observation of our patient, whenever he assumes the horizontal posture, from the circumstance that one of the inferior extremities is not perfectly extended upon the body, although he may be perfectly unconscious of the fact?

In concluding this paper, the writer feels assured that he has been true to his prediction in the outset, that he has added nothing to the diagnosis or treatment of the disease alluded to; but if the meagre investigation which he has attempted of the subject, shall serve, in the least, to invite the acumen and patient industry of some one more competent to do justice to so ample a field of labor, than himself, he will feel most liberally rewarded, and that his whole purpose has been fully accomplished.

